# Effect of direct oral anticoagulant dabigatran on early bone healing: An experimental study in rats

**DOI:** 10.34172/japid.2023.020

**Published:** 2023-11-07

**Authors:** Ioanna Kyriakaki, Theodora Karanikola, Theodoros Lillis, Eleana Kontonasaki, Nikolaos Dabarakis

**Affiliations:** ^1^Department of Dentoalveolar Surgery, Surgical Implantology and Roentgenology, Aristotle University, Thessaloniki, Greece; ^2^Private Practice, Clinical Instructor, Department of Oral Surgery, Implantology and Dental Radiology, School of Dentistry, Faculty of Health Sciences, Aristotle University of Thessaloniki, Thessaloniki, Greece; ^3^Department of Prosthodontics, School of Dentistry, Aristotle University of Thessaloniki, Greece

**Keywords:** Anticoagulants, Bone regeneration, Dabigatran, Parietal bone, Wistar rats

## Abstract

**Background.:**

Dabigatran belongs to the new generation of direct oral anticoagulants (DOACs). Its advantages are oral administration and no need for international normalized ratio (INR) monitoring. Although its use has increased, its potential side effects on bone healing and remodeling have not been fully investigated. The present study aimed to evaluate the possible effects of dabigatran on early bone healing.

**Methods.:**

Sixteen male Wistar rats were divided into two groups; in group A, 20-mg/kg dabigatran dose was administered orally daily for 15 days, while group B served as a control. Two circular bone defects (d=6 mm) were created on either side of the parietal bones. Two weeks after surgery and euthanasia of the animals, tissue samples (parietal bones that contained the defects) were harvested for histological and histomorphometric analysis. Statistical analysis was performed with a significance level of α=0.5.

**Results.:**

No statistically significant differences were found between the two groups regarding the regenerated bone (21.9% vs. 16.3%, *P*=0.172) or the percentage of bone bridging (63.3% vs. 53.5%, *P*=0.401).

**Conclusion.:**

Dabigatran did not affect bone regeneration, suggesting that it might be a safer drug compared to older anticoagulants known to lead to bone healing delay.

## Introduction

 The demand for anticoagulant medications has increased as thrombosis remains a major source of morbidity and mortality associated with multiple diseases such as strokes, pulmonary embolism, deep vein thrombosis, etc.^1,2^ For many years, anticoagulant drugs included vitamin K antagonists (acenocoumarol, warfarin, etc.) and/or heparins, while better understanding of coagulation cascade events at a molecular level and pharmacokinetics of anticoagulant substances have led to the design of a new generation of anticoagulants.^3^ Vitamin K antagonists have recently been replaced with direct oral anticoagulants (DOACs) and, compared to warfarin, have shown the same or greater efficacy and safety.^4^ Warfarin administration requires regular monitoring of international normalized ratio (INR) due to its small therapeutic window and significant variability among patients in dosage response.^5,6^ DOACs inhibit specific clotting factors, unlike warfarin, which affects several vitamin K-dependent coagulation factors.^7,8^ Direct thrombin inhibitors (dabigatran) and factor Xa inhibitors (rivaroxaban, apixaban, and edoxaban) are the most common DOACs.^3^ DOACs are delivered orally and do not interfere with the cycle of vitamin K. Therefore, their effect is not affected by diet and does not cause osteopenia or vascular calcification. Dabigatran is a competitive, direct thrombin inhibitor. It inhibits both free and fibrin-bound thrombin, unlike heparin, which only manages to bind to free thrombin.^9^ Dabigatran prolongs coagulation markers such as the activated partial thromboplastin time, ecarin clotting time, thrombin time, and dilute thrombin time, but not INR.^10-13^ It is administered orally; however, it is not absorbed from the gastrointestinal tract tube in this form but as a prodrug (dabigatran etexilate).^9^ Dabigatran etexilate is rapidly absorbed and then converted to dabigatran through hydrolysis in the liver and plasma by esterase as a catalyst.^14^ In recent years, the use of DOACs has grown significantly for the prevention of thromboembolic events, and many dental and maxillofacial surgeons now must handle patients who are taking such drugs and require oral surgical interventions.^15^ Controversy exists about whether DOACs should be discontinued for 24 hours when teeth extractions or dental implant placement are needed.^16,17^ However, a recent systematic review and meta-analysis has revealed that if local hemostatic precautions are taken, continuing DOACs therapy does not increase the risk of bleeding in patients having undergone implant surgery.^18^ Similarly, Gómez-Moreno et al^19^ suggested that patients receiving dabigatran therapy can safely undergo dental implant surgery provided that the last dose is administered 12 hours before and the next one is administered not less than 8 hours after surgery. Based on the literature findings, it seems that until further solid evidence is achieved, before performing dental implant surgery, the physician must weigh the risks and benefits of stopping DOACs while taking into account patient and surgical considerations.^20^ Bone regeneration after a trauma is a complex and well-orchestrated sequence of cellular and molecular events that lead to the reconstruction of the damaged area and restoration of its functionality.^21^ As a first step, the hemostasis process begins with an accumulation of blood in the fracture area and clot formation. The sequence of events leading to hemostasis includes active coagulation factors, as well as many molecular factors derived from the periosteum, bone marrow, platelets, and surrounding soft tissues.^22^ These factors include cytokines, growth factors (vascular endothelial growth factor), the transforming growth factor β1, morphogenetic proteins, and factors related to angiogenesis and osteogenesis. Inside the clot, activated platelets are connected to each other by fibrin fibers, creating a fibrin network, in which various components, such as erythrocytes and leukocytes, are often trapped.^23^ This network acts as a scaffold for osteogenesis and is created when the extrinsic coagulation pathway is activated.^23,24^ Clotting factors, activated platelets, and other blood cells all play crucial roles in the activation of this pathway.^23,25^ Activated platelets keep the clot in place, while polyphosphatases modulate pore size and remodel the fibrillar network. Other cells, such as fibroblasts, leukocytes, and endothelial cells secrete factors that regulate thrombin production. Thrombin is a trypsin-like serine protease that plays a key role in the coagulation cascade.^26^ Thrombin leads to the detachment of part of the fibrinogen and turns it into fibrin, while the fibrin polymerization process subsequently begins. Growth factors bind to the fibrin and thus trigger the initiation of bone healing.^23,25^ During bone repair, progenitor cells are recruited, and their proliferation and differentiation into osteoblasts and osteoclasts governs subsequent bone formation.^23,25,27-29^

 The role of thrombin and its deficiency on bone microstructure and bone density has been investigated in a few studies, and the involved mechanisms have not been fully elucidated. Thrombin has been reported to promote interleukin 6 (IL-6) and prostaglandin E2 (PGE2) expression, favoring osteoclast activation and demineralization of bone matrix by increased expression of RANKL relative to OPG.^30,31^ According to Tudpor et al,^32^ thrombin receptor impairment causes a drop in the RANKL/OPG ratio, which is linked to a high bone density phenotype. Sivagurunathan et al^33^ reported that osteoclast differentiation is inhibited by thrombin, which exerts anabolic effects on osteoblastic lineage cells. Both anticoagulants and antiplatelet drugs can interfere with clot formation, exercising antithrombotic activity with different mechanisms. Their role in bone healing and fracture risk has mainly been investigated with controversial findings.^34-39^

 As dabigatran is a direct thrombin inhibitor, it would be interesting to investigate its effects on bone formation and healing after trauma, as there is limited literature showing either potential positive or negative effects on bone formation. Thus, the present study aimed to investigate early bone healing of calvaria bone defects of rats receiving dabigatran and its possible effects on bone regeneration.

## Methods

###  Animal study design 

 Sixteen male Wistar rats aged 2‒3 months with a mean weight of 360.4 g were used in the study. Animal selection, management, and surgery protocol were approved by the Ethics Committee of the Dental School, Aristotle University of Thessaloniki (168139/1229). The animals were fed ad libitum with standard laboratory food pellets during the experiment.

 The animals were randomly divided into an experimental (dabigatran) group and a control group (n = 8). In the experimental group, dabigatran (20 mg/kg) was delivered daily for 15 days, starting from 24 hours before the surgical procedure. The dose was chosen after literature research, which revealed many different amounts of doses, with significant variations from 10 mg/kg to 50 mg/kg. An intermediate dose was randomly chosen. Dabigatran capsules were smashed and weighed on a high-accuracy balance to prepare daily doses. As dabigatran is absorbed from the gastrointestinal tract, it was delivered orally. In the control group, no intervention was made.

###  Surgical procedures 

**Figure 1:**
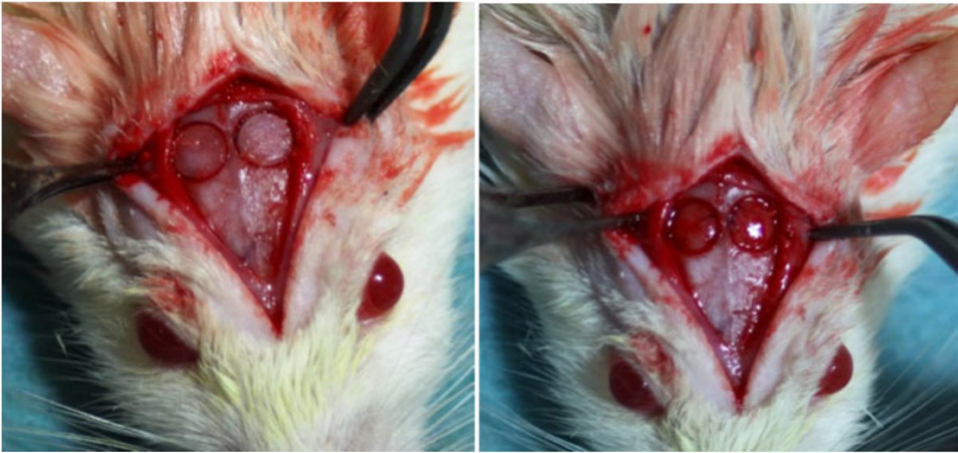
Pictures of the calvaria defects on rats’ parietal bones

 Every animal received antibiotic prophylaxis (Begalin-P PD injection (Sol; Pfizer Hellas, 50 mg/kg subcutaneously) 1 hour before the general anesthesia and after the surgery. The surgical procedure took place 24 hours after the delivery of dabigatran. For general anesthesia, the animals were given ketamine (40‒100 mg/kg, i.m., Imalgene, Merial, France, and xylazine 2‒5 mg/kg intramuscularly). Before surgery, the dorsal part of the rat calvarium was shaved, and the skin was disinfected using a 10% polyvidone iodine solution. A median sagittal incision was made along the top of the skull, parietal bones were exposed, and two circular calvarial bone defects, 1‒2 mm in thickness, were created on both sides of the sagittal suture, with a 6-mm-diameter trephine drill at 1500 rpm under saline irrigation to prevent excessive heating (Figure 1).^40^ According to the study of Porto et al^41^ in 2012, this size of the defects is critical for the 15-day evaluation period. Then, the periosteum and skin were carefully sutured with 4-0 silk (skin) and 4-0 vicryl (periosteum). After the operation, the animals were kept in separate cages following the same diet. There was a complete daily post-surgery follow-up of weight and status.

 Fifteen days after the operation, the animals were anesthetized and euthanized by an intravenous administration of pentobarbital (18% solution, 60 mg/kg). Following their euthanasia, whole-body perfusion fixation with 10% neutral buffered formalin was performed, and the cranial bone areas containing the defects were block-sectioned for histological preparation and histomorphometry analysis.

###  Histological preparation 

 All the specimens were initially immersed in a 10% formaldehyde solution for fixation, followed by dehydration by sequential immersion of ascending concentrations of alcohol. Subsequently, the specimens were infiltrated by methylmethacrylate by immersion in alcoholic solutions of increasing concentrations of methylmethacrylate. Next, 80-μm methylmethacrylate-embedded tissue sections were prepared for histological evaluation using the EXAKT system (Advanced Technologies GmbH, Norderstedt, Germany). The specimens were cut vertically, and histological sections were duly oriented to coincide with the direction of the defect diameter. The sections were then conventionally stained with toluidine blue/basic fuchsin.

###  Histomorphometry 

**Figure 2:**
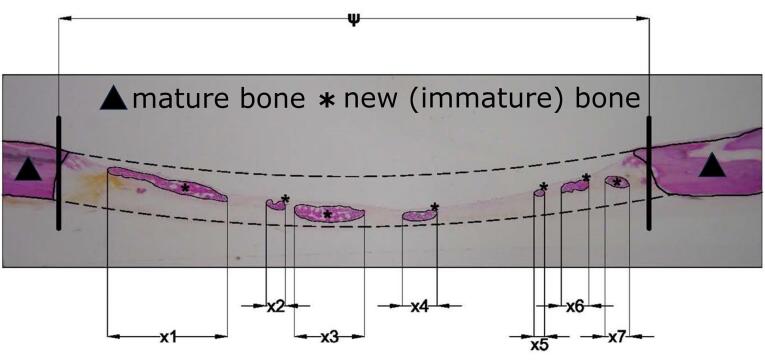
Schematic representation of calculated areas for bone formation on optical microscope images. (ψ: total length of the defect, x1 + x2 + … + xn: length of bridging with newly formed bone)

 The samples were viewed under an optical microscope (Zeiss Axio Lab, Germany), and the development and degree of the new bone maturation were recorded. Digital images were captured (SONY DSC F707) to perform histomorphometry measurements using the special software Image Pro Plus (Media Cybernetics Inc., Rockville, MD, United States). With the above program, it was calculated (Figure 2):

 A. The percentage (%) of bone defect regeneration: the surface of newly formed bone/total surface of the defect × 100%

 B. The percentage (%) of defect bridging: the length of bridging with newly formed bone/ total length of the defect × 100%

###  Sample size calculation 

 Based on our pilot study,^40^ we considered that the primary outcome (defect regeneration percentage) in the control group would be approximately 20 ± 10%. Based on this, to detect a difference of ± 15% between the groups, at least seven animals would be needed in each group to reach a power (1-β) of 0.80, with α = 0.05. G*Power v.3.1.9.2 (Frantz Faul, Univerisität Kiel, Germany) was used to calculate the sample size.

###  Statistical analysis 

 Statistical analyses were performed using SPSS 20.0 (SPSS, Inc., Chicago, IL, USA). The average percentages of bone regeneration and defect bridging were initially calculated from the two defects for each animal. The histomorphometric parameters were presented as mean ± standard deviation within the groups. The significance of differences between the groups was determined by the t-test for independent samples since the data met the criteria for normal distribution, as indicated by the Shapiro-Wilk test. Statistical significance was determined at *P* < 0.05.

## Results

**Figure 3:**
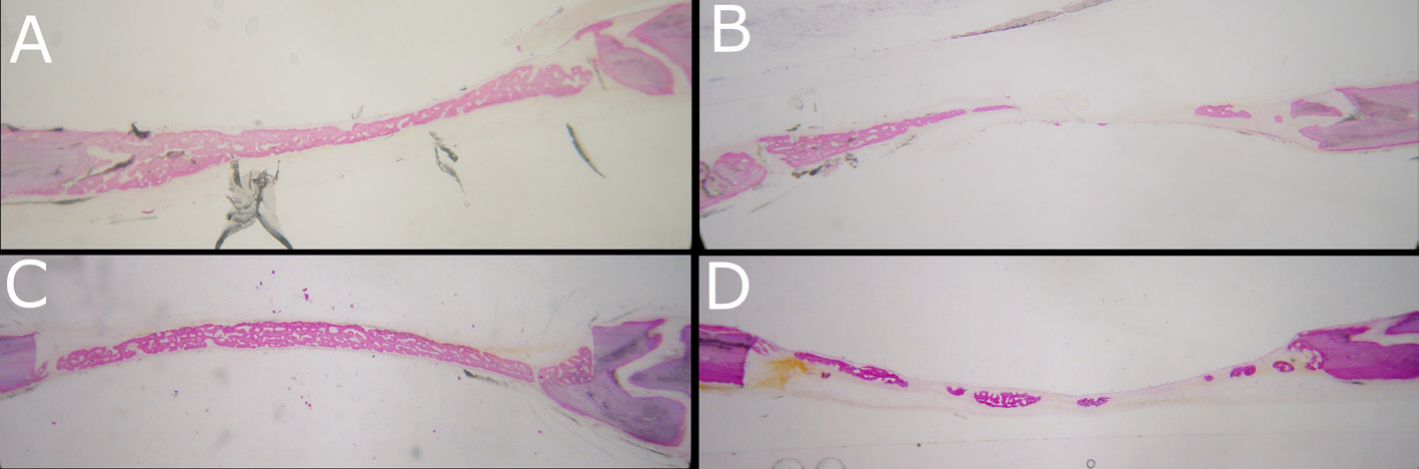
Representative images of bone regeneration. (A) Complete bridging (dabigatran group), (B) Partial bridging (dabigatran group), (C) Complete bridging (control group), (D) Islets of woven bone (control group)

 Surgical procedures were completed without complications, and all the animals recovered well from the sedation and interventions. The postoperative period was uneventful, and all the animals completed the study without any bleeding complications, surgical wound dehiscence, signs of infection, or other complications. Macroscopic postmortem inspection showed that none of the defects was completely regenerated. In both groups, histological examination showed partial coverage of defects with newly formed bone, mostly woven (Figure 3). A complete bridging of the defect was noticed in five histological specimens of the dabigatran group (Figure 3A) and in seven histological specimens of the control group (Figure 3C), possibly caused by the remaining periosteum in the wound area. In the rest of the specimens, either the bridging was not complete (Figure 3B), or small islets of newly formed bone existed in the center of the defect, indicating osteogenesis coming from the periosteum and the dura mater (Figure 3D). Between the two groups, there was no statistically significant difference either in the percentage of newly formed bone or in the percentage of defect bridging (Table 1).

**Table 1:** Meanpercentages (%) of bone regeneration and defect bridging between groups

	**Dabigatran group**	**Control group**	***P***** value**
Bone regeneration (%)	21.9 ± 6.0	16.3 ± 11.2	0.172
Defect bridging (%)	63.3 ± 14.5	53.5 ± 22.3	0.401

## Discussion

 To the best of our knowledge, the present study is the first to investigate the role of dabigatran in early bone healing in rat calvarial bone defects. The results showed that systematic delivery of dabigatran did not affect bone regeneration, consistent with a similar study by Kerimoglu et al,^11^ in which the authors examined the effect of dabigatran in tibial fractures in rats. In their study, four groups received different doses of dabigatran with various delivery durations. (Group 1: 10 mg/kg for 14 days, group 2: 10 mg/kg for 28 days, group 3: 50 mg/kg for 14 days, group 4: 50 mg/kg for 28 days). Their study showed no statistically significant difference between the groups receiving dabigatran for 14 (groups 1 and 3) or 28 days (groups 2 and 4) regarding the radiologic or histomorphometric evaluations. Therefore, the drug dose seems not to affect the outcomes. However, there was a statistically significant difference when the groups were compared regarding the duration of drug delivery (comparison between groups 1‒2 and 3‒4), which underlines the possible effect of the delivery duration.

 Moreover, Fusaro et al^10^ compared the effect of dabigatran related to warfarin administration on bone structure and vascular calcification in rats. The animals were divided into three groups: the first as normal control (untreated), the second with delivery of dabigatran (1 mg/g of food, 15‒30 g a day in total), and the third with delivery of warfarin in a dose to reach a concentration sufficient to obtain an INR between 2 and 3. After sacrificing the animals, the femur, tibia, and vertebrae were collected and stored in ethanol for immunohistochemical and morphometric analyses of bone remodeling. In the warfarin group, a histomorphometric study of the femur and vertebrae revealed dramatically reduced bone volume and increased trabecular separation. Vertebral examination revealed that the rats receiving dabigatran had more trabecular tissue. Except for maximum erosion depth, which was higher in warfarin-treated rats, possibly indicating increased osteoclastic activity, osteoblast activity and resorption parameters were comparable between the groups. As a result, warfarin was linked to increased bone formation and activation frequency, possibly leading to increased bone remodeling with higher osteoclast activity. Rats treated with warfarin had lower bone volume, greater trabecular separation, and higher turnover than those treated with dabigatran or the control group. These findings imply that compared to warfarin, dabigatran has a higher bone safety profile. These variations may translate into a decreased incidence of fractures in dabigatran-treated individuals since warfarin medication impacts bone by diminishing trabecular size and structure, increasing turnover, and reducing mineralization. Similar to dabigatran, the production of massive calluses and an increase in bone mineral density reported in a rat model of femur fracture suggest that rivaroxaban (factor Xa inhibitor) may beneficially affect fracture healing.^42^

 Brent et al^43^ used male and female C57BL/6 mice and evaluated the role of dabigatran mixed in chow in bone mineral density and bone mineral content (BMC) of various murine bones. They concluded that despite the relatively large dose of dabigatran utilized (1.52 and 1.70/g body weight for females and males, respectively), neither male nor female mice exhibited any significant detrimental effects on bone tissue, apart from a small favorable site-specific effect at the tibial cortical bone in female mice.

 Numerous studies have tried to elaborate on the effect of DOACs on increased fracture risk or new-onset osteoporosis. However, although they have been found superior compared to vitamin K antagonist (VKA) anticoagulants,^42,44-46^ there are no clear findings on whether differences exist between the different classes of DOACs^47-50^ and differences exist between studies. In a recent network meta-analysis with osteoporotic fractures, including 321 844 patients with a follow-up of two years, it was found that from all the DOACs currently on the market, apixaban has the lowest likelihood of developing an osteoporotic fracture.^50^ Another population-based cohort study^48^ found that when opposed to taking warfarin, patients with atrial fibrillation who utilize DOACs may experience a lower risk of osteoporotic fracture; however, the kind of DOAC does not appear to change the fracture risk.

 Concerning other new generations of anticoagulants, a few studies have evaluated their effect on bone. Xia et al^51^ compared the effect of heparin with rivaroxaban on rats and examined the levels of calcium and phosphorus in serum, markers of bone formation (e.g., alkaline phosphatase and PINP), and markers of bone resorption (pyridinoline and deoxypyridinoline) for assessing bone metabolism. Additionally, energy x-ray absorptiometry and a CT scan were used to compare trabecular and cortical bone microstructures. The serum calcium and phosphorus levels were comparable between the heparin and rivaroxaban groups, but the markers of bone formation and bone resorption differed. The group that received heparin showed higher bone resorption markers but lower activity and levels of bone formation markers. Rivaroxaban, on the other hand, only caused PINP levels to drop. Heparin hindered bone growth and accelerated bone resorption, according to the study’s findings. Both trabecular and cortical bone morphometric parameters were impacted by heparin. Cortical volume was decreased in rats receiving heparin treatment, according to micro-CT studies of cortical bones. However, following rivaroxaban therapy, no appreciable change was observed. The researchers concluded that rivaroxaban had less detrimental effects on bone microstructure than heparin. Klüter et al^52^ evaluated the effect of administrating rivaroxaban at a dose of 3 mg/kg body weight per day for 28 and 49 days after creating femur fractures on Wistar rats. They concluded that rivaroxaban did not impair fracture healing, although they emphasized the small number of animals in their study.

 Significant findings can be achieved from in vitro studies employing DOACs and other anticoagulants in different bone cells, although here also, the results are controversial. Rocha et al^53^ evaluated the effect of dabigatran (Pradaxa® capsule, Boehringer, Ingelheim am Rhein, Germany) on different cell cultures, including osteoclasts. Although heparin effects have been documented on osteoclasts,^54^ data on DOAC effects are not yet available. They used bone marrow-derived osteoclasts isolated from the femurs and tibiae of C57BL/6 mice, osteoblasts derived from calvaria fragments of newborn Wistar Hannover rats, and a pre-osteoblastic cell line (American Type Culture Collection, Manassas, VA, USA). They reported reduced osteoclast differentiation at the highest tested concentrations (2 μg/mL and 3 μg/mL), as verified by TRAP staining and downregulation of CTSK expression, which is a key marker of osteoclast differentiation and activity. They also reported reduced osteoblast differentiation, as confirmed by reduced alkaline phosphatase activity and mineralized matrix formation. They also administered 428.5 μL of an aqueous solution of dabigatran etexilate at 100 μg/mL concentration twice daily in 5 rats for 28 days and investigated whether their BMCs retained their ability to differentiate into osteoclasts. They found that despite no significant differences in the TRAP-stained osteoclasts, their resorption capacity was significantly restrained.

 Opposite results were found in an in vitro study by Winkler et al.^27^ The study aimed to investigate the effect of melagatran, a direct thrombin inhibitor, on human osteoblasts. Osteoblast cultures were derived from cancellous bone from 6 individuals, harvested during total knee replacement. Melagatran, dalteparin, and unfractionated heparin (UFH) were added to primary osteoblast cultures. Cell number, protein synthesis, mitochondrial and alkaline phosphatase activity, and collagen type I synthesis were evaluated. In the highest investigated concentration, melagatran only reduced the cell count to 84% of the control group after 15 days of incubation. In contrast, the reduction of cell count was far more pronounced under the influence of dalteparin (39%) and UFH (10%). Melagatran showed less inhibitory in vitro effects on human osteoblasts than dalteparin or UFH.

 In vitro effects of other DOACs have shown that rivaroxaban can inhibit the proliferation of female-derived osteoblasts. Gigi et al^55^ investigated the direct effects of rivaroxaban on bone biology; the in vitro model demonstrated that osteoblastic mineralization was unaffected. The study’s findings indicated that rivaroxaban inhibits the first stage of bone formation but does not affect later stages (i.e., bone mineralization).

 The present study had some limitations. For example, the evaluation of bone healing at different time intervals was not conducted, nor was the administration of different doses of dabigatran for different perioperative periods. However, since dabigatran affects the coagulation cascade, it may be hypothesized that any effect would occur in the early stages of the defect healing. Moreover, our study would have benefited from using micro-computed tomography to obtain 3-D quantitative data of the defect regeneration.

## Conclusion

 Under the limitations of the present study, systematic delivery of dabigatran seems not to affect bone regeneration in calvarial defects in rats. There were no statistically significant differences between the two groups, the control and the one with dabigatran administration, either in the percentage of newly formed bone or in the percentage of defect bridging. However, the findings were slightly better for the dabigatran group. This finding adds another benefit to using the new generation of coagulants. However, further studies are needed, both in vitro and in vivo, to elucidate the underlying mechanisms of bone healing in patients receiving DOACs, as from the limited literature, it seems that gender, duration, and administration dose may be factors that can play a significant role. Further in vivo studies should involve multiple DOACs to clarify which factors may contribute to the different results obtained with the various types of drastic DOAC substances.

## Competing Interests

 The authors declare that they have no financial and non-financial competing interests concerning the publication of their work during submission.

## Data Availability Statement

 The data produced during the present study are available from the corresponding author upon reasonable request.

## Ethical Approval

 The present study was approved by the Protocol Approval Committee of the Directorate of Veterinary Medicine of the Region of Central Macedonia for implementation of rules and ethics during experimentation on laboratory animals in accordance with Presidential Decree 56/2013, at its meeting on June 30, 2017, with the protocol number 168139/1229.

## Funding

 This research received no specific grant from funding agencies in the public, commercial, or not-for-profit sectors.

## References

[1] Kapil N, Datta YH, Alakbarova N, Bershad E, Selim M, Liebeskind DS (2017). Antiplatelet and anticoagulant therapies for prevention of ischemic stroke. Clin Appl Thromb Hemost.

[2] Kwaan HC, Samama MM (2004). Anticoagulant drugs: an update. Expert Rev Cardiovasc Ther.

[3] Mega JL, Simon T (2015). Pharmacology of antithrombotic drugs: an assessment of oral antiplatelet and anticoagulant treatments. Lancet.

[4] Wu X, Cao S, Yu B, He T (2022). Comparing the efficacy and safety of direct oral anticoagulants versus vitamin K antagonists in patients with antiphospholipid syndrome: a systematic review and meta-analysis. Blood Coagul Fibrinolysis.

[5] Ansell J, Hirsh J, Poller L, Bussey H, Jacobson A, Hylek E (2004). The pharmacology and management of the vitamin K antagonists: the Seventh ACCP Conference on Antithrombotic and Thrombolytic Therapy. Chest.

[6] Hirsh J, O’Donnell M, Eikelboom JW (2007). Beyond unfractionated heparin and warfarin: current and future advances. Circulation.

[7] Wilson MR, Docherty KF, Gardner RS (2016). Use of direct oral anticoagulants in thromboembolic disease. Prescriber.

[8] van Gorp RH, Schurgers LJ (2015). New insights into the pros and cons of the clinical use of vitamin K antagonists (VKAs) versus direct oral anticoagulants (DOACs). Nutrients.

[9] Ganetsky M, Babu KM, Salhanick SD, Brown RS, Boyer EW (2011). Dabigatran: review of pharmacology and management of bleeding complications of this novel oral anticoagulant. J Med Toxicol.

[10] Fusaro M, Dalle Carbonare L, Dusso A, Arcidiacono MV, Valenti MT, Aghi A (2015). Differential effects of dabigatran and warfarin on bone volume and structure in rats with normal renal function. PLoS One.

[11] Kerimoglu S, Onay A, Guvercin Y, Çitlak A, Yenilmez E, Kerimoglu G (2015). The effects of dabigatran etexilate on fracture healing in rats: an experimental study. Indian J Orthop.

[12] Liakoni E, Rätz Bravo AE, Krähenbühl S (2015). Hepatotoxicity of new oral anticoagulants (NOACs). Drug Saf.

[13] Bunchorntavakul C, Reddy KR (2017). Drug hepatotoxicity: newer agents. Clin Liver Dis.

[14] Blech S, Ebner T, Ludwig-Schwellinger E, Stangier J, Roth W (2008). The metabolism and disposition of the oral direct thrombin inhibitor, dabigatran, in humans. Drug Metab Dispos.

[15] Lanau N, Mareque J, Giner L, Zabalza M (2017). Direct oral anticoagulants and its implications in dentistry A review of literature. J Clin Exp Dent.

[16] Buchbender M, Schlee N, Kesting MR, Grimm J, Fehlhofer J, Rau A (2021). A prospective comparative study to assess the risk of postoperative bleeding after dental surgery while on medication with direct oral anticoagulants, antiplatelet agents, or vitamin K antagonists. BMC Oral Health.

[17] Bajkin BV, Wahl MJ, Miller CS (2020). Dental implant surgery and risk of bleeding in patients on antithrombotic medications: a review of the literature. Oral Surg Oral Med Oral Pathol Oral Radiol.

[18] Zou L, Hua L (2023). Risk of bleeding with dental implant surgery in patients on anticoagulant or antiplatelet drugs: a systematic review and meta-analysis. Acta Odontol Scand.

[19] Gómez-Moreno G, Fernández-Cejas E, Aguilar-Salvatierra A, de Carlos F, Delgado-Ruiz RA, Calvo-Guirado JL (2018). Dental implant surgery in patients in treatment by dabigatran. Clin Oral Implants Res.

[20] Dawoud BES, Kent S, Tabbenor O, George P, Dhanda J (2021). Dental implants and risk of bleeding in patients on oral anticoagulants: a systematic review and meta-analysis. Int J Implant Dent.

[21] Söhling N, Von Jan O, Janko M, Nau C, Ritz U, Marzi I (2023). Measuring bone healing: parameters and scores in comparison. Bioengineering (Basel).

[22] Periayah MH, Halim AS, Mat Saad AZ (2017). Mechanism action of platelets and crucial blood coagulation pathways in hemostasis. Int J Hematol Oncol Stem Cell Res.

[23] Wang X, Friis T, Glatt V, Crawford R, Xiao Y (2017). Structural properties of fracture haematoma: current status and future clinical implications. J Tissue Eng Regen Med.

[24] Schell H, Duda GN, Peters A, Tsitsilonis S, Johnson KA, Schmidt-Bleek K (2017). The haematoma and its role in bone healing. J Exp Orthop.

[25] Mathavan N, Turunen MJ, Guizar-Sicairos M, Bech M, Schaff F, Tägil M (2018). The compositional and nano-structural basis of fracture healing in healthy and osteoporotic bone. Sci Rep.

[26] Di Cera E (2008). Thrombin. Mol Aspects Med.

[27] Winkler T, Perka C, Matziolis D, Matziolis G (2011). Effect of a direct thrombin inhibitor compared with dalteparin and unfractionated heparin on human osteoblasts. Open Orthop J.

[28] Bigham-Sadegh A, Oryan A (2015). Selection of animal models for pre-clinical strategies in evaluating the fracture healing, bone graft substitutes and bone tissue regeneration and engineering. Connect Tissue Res.

[29] Wang X, Wang Y, Gou W, Lu Q, Peng J, Lu S (2013). Role of mesenchymal stem cells in bone regeneration and fracture repair: a review. Int Orthop.

[30] Braun T, Zwerina J (2011). Positive regulators of osteoclastogenesis and bone resorption in rheumatoid arthritis. Arthritis Res Ther.

[31] Okada Y, Lorenzo JA, Freeman AM, Tomita M, Morham SG, Raisz LG (2000). Prostaglandin G/H synthase-2 is required for maximal formation of osteoclast-like cells in culture. J Clin Invest.

[32] Tudpor K, van der Eerden BC, Jongwattanapisan P, Roelofs JJ, van Leeuwen JP, Bindels RJ (2015). Thrombin receptor deficiency leads to a high bone mass phenotype by decreasing the RANKL/OPG ratio. Bone.

[33] Sivagurunathan S, Pagel CN, Loh LH, Wijeyewickrema LC, Pike RN, Mackie EJ (2013). Thrombin inhibits osteoclast differentiation through a non-proteolytic mechanism. J Mol Endocrinol.

[34] Syberg S, Brandao-Burch A, Patel JJ, Hajjawi M, Arnett TR, Schwarz P (2012). Clopidogrel (Plavix), a P2Y12 receptor antagonist, inhibits bone cell function in vitro and decreases trabecular bone in vivo. J Bone Miner Res.

[35] Mediero A, Wilder T, Reddy VS, Cheng Q, Tovar N, Coelho PG (2016). Ticagrelor regulates osteoblast and osteoclast function and promotes bone formation in vivo via an adenosine-dependent mechanism. FASEB J.

[36] Jørgensen NR, Grove EL, Schwarz P, Vestergaard P (2012). Clopidogrel and the risk of osteoporotic fractures: a nationwide cohort study. J Intern Med.

[37] Lillis T, Veis A, Sakellaridis N, Tsirlis A, Dailiana Z (2019). Effect of clopidogrel in bone healing-experimental study in rabbits. World J Orthop.

[38] Tsai SHL, Hu CW, Shao SC, Tischler EH, Obisesan OH, Vervoort D (2022). Comparative risks of fracture among direct oral anticoagulants and warfarin: a systematic review and network meta-analysis. Front Cardiovasc Med.

[39] Lindner T, Cockbain AJ, El Masry MA, Katonis P, Tsiridis E, Schizas C (2008). The effect of anticoagulant pharmacotherapy on fracture healing. Expert Opin Pharmacother.

[40] Karanikola T, Cheva A, Sarafidou K, Myronidou-Tzouveleki M, Tsavdaridis I, Kontonasaki E (2022). Effect of diclofenac and simvastatin on bone defect healing-an in vivo animal study. Biomimetics (Basel).

[41] Porto GG, Vasconcelos BC, Andrade ES, Carneiro SC, Frota MS (2012). Is a 5 mm rat calvarium defect really critical?. Acta Cir Bras.

[42] Gu ZC, Zhou LY, Shen L, Zhang C, Pu J, Lin HW (2018). Non-vitamin K antagonist oral anticoagulants vs warfarin at risk of fractures: a systematic review and meta-analysis of randomized controlled trials. Front Pharmacol.

[43] Brent MB, Thomsen JS, Brüel A (2018). The effect of oral dabigatran etexilate on bone density, strength, and microstructure in healthy mice. Bone Rep.

[44] Patil T, Hobson J (2021). Risk of new-onset osteoporosis in single-center veteran population receiving direct oral anticoagulants versus warfarin. Thromb Res.

[45] Huang HK, Peng CC, Lin SM, Munir KM, Chang RH, Wu BB (2021). Fracture risks in patients treated with different oral anticoagulants: a systematic review and meta-analysis. J Am Heart Assoc.

[46] Yokoyama S, Ieda S, Nagano M, Nakagawa C, Iwase M, Hosomi K (2020). Association between oral anticoagulants and osteoporosis: real-world data mining using a multi-methodological approach. Int J Med Sci.

[47] Lutsey PL, Norby FL, Ensrud KE, MacLehose RF, Diem SJ, Chen LY (2020). Association of anticoagulant therapy with risk of fracture among patients with atrial fibrillation. JAMA Intern Med.

[48] Lau WCY, Cheung CL, Man KKC, Chan EW, Sing CW, Lip GYH (2020). Association between treatment with apixaban, dabigatran, rivaroxaban, or warfarin and risk for osteoporotic fractures among patients with atrial fibrillation: a population-based cohort study. Ann Intern Med.

[49] Huang HK, Liu PP, Hsu JY, Lin SM, Peng CC, Wang JH (2020). Risk of osteoporosis in patients with atrial fibrillation using non-vitamin K antagonist oral anticoagulants or warfarin. J Am Heart Assoc.

[50] Khanra D, Mukherjee A, Deshpande S, Khan H, Kathuria S, Kella D (2021). A network meta-analysis comparing osteoporotic fracture among different direct oral anticoagulants and vitamin K antagonists in patients with atrial fibrillation. J Bone Metab.

[51] Xia J, Zhang Z, Wang J, Zu J, Wang N, Wang D (2015). Comparison of the effects of heparin and the direct factor Xa inhibitor, rivaroxaban, on bone microstructure and metabolism in adult rats. Connect Tissue Res.

[52] Klüter T, Weuster M, Brüggemann S, Menzdorf L, Fitschen-Oestern S, Steubesand N (2015). Rivaroxaban does not impair fracture healing in a rat femur fracture model: an experimental study. BMC Musculoskelet Disord.

[53] Rocha AL, Bighetti-Trevisan RL, Duffles LF, de Arruda JAA, Taira TM, Assis BRD (2020). Inhibitory effects of dabigatran etexilate, a direct thrombin inhibitor, on osteoclasts and osteoblasts. Thromb Res.

[54] Folwarczna J, Sliwiński L, Janiec W, Pikul M (2005). Effects of standard heparin and low-molecular-weight heparins on the formation of murine osteoclasts in vitro. Pharmacol Rep.

[55] Gigi R, Salai M, Dolkart O, Chechik O, Katzburg S, Stern N (2012). The effects of direct factor Xa inhibitor (rivaroxaban) on the human osteoblastic cell line SaOS2. Connect Tissue Res.

